# Sweet-Syndrom des Kindesalters mit erworbener Cutis laxa (Marshall-Syndrom) als Erstmanifestation einer Takayasu-Arteriitis

**DOI:** 10.1007/s00105-022-04999-2

**Published:** 2022-07-15

**Authors:** Christiane Michl, Regina Hühn, Cord Sunderkötter

**Affiliations:** 1 Universitätsklinik und Poliklinik für Dermatologie und Venerologie, Universitätsklinikum Halle/Saale, Halle/Saale, Deutschland; 2 Universitätsklinik und Poliklinik für Dermatologie und Venerologie, Ernst-Grube-Str. 40, 06120 Halle, Deutschland; 3 Universitätsklinik und Poliklinik für Pädiatrie I, Universitätsklinikum Halle/Saale, Halle/Saale, Deutschland

**Keywords:** Neutrophile Dermatosen, Kardiovaskuläre Beteiligung, Aortitis, Vaskulitis, Tozilicumab, Neutrophilic dermatosis, Cardiovascular involvement, Aortitis, Vasculitis, Tozilizumab

## Abstract

Eine Besonderheit des seltenen Sweet-Syndroms des Kindesalters ist die fakultative Abheilung in Form einer postinflammatorischen Elastolyse mit erworbener Cutis laxa, die nach dem Erstbeschreiber als Marshall-Syndrom benannt wird. Wir berichten von einem 3‑jährigen Kind, bei dem ein derartiges Sweet-Syndrom zur Erstdiagnose einer Takayasu-Arteriitis führte. Die postinflammatorische Elastolyse mit erworbener Cutis laxa stellt beim kindlichen Sweet-Syndrom einen klinisch relevanten kutanen Indikator für lebensbedrohliche kardiale Gefäßkomplikationen wie Aortitis, Aortenaneurysma, Koronararterienstenose und Herzversagen dar. Da das Cutis-laxa-artig abheilende Sweet-Syndrom den kardialen Komplikationen zumeist zeitlich vorausgeht oder wie in unserem Fall simultan auftritt, sollten die betroffenen Patienten umgehend kardiologisch und rheumatologisch untersucht werden, um bei vaskulärer Beteiligung einen komplikativen Verlauf durch frühe antiinflammatorische und immunmodulierende Systemtherapie zu verhindern.

## Abstract (en)

A special form of the rare infantile Sweet syndrome (acute febrile neutrophilic dermatosis) is facultative healing in the form of postinflammatory elastolysis with acquired cutis laxa, named “Marshall” syndrome after the authors who first described it. We report the case of a 3-year-old child in whom the cutaneous manifestation led to diagnosis of Takayasu arteritis. Postinflammatory elastolysis with acquired cutis laxa is a clinically relevant cutaneous indicator of life-threatening cardiovascular complications such as aortitis, aortic aneurysm, coronary stenosis and heart failure in children with Sweet’s syndrome. Cutis laxa usually precedes cardiac complications or, as in our case, occurs simultaneously; thus, immediate cardiac and rheumatologic examinations are important to initiate systemic therapy with anti-inflammatory and immunomodulatory agents early to prevent complications.

Das Sweet-Syndrom des Kindesalters mit postinflammatorischer Elastolyse und erworbener Cutis laxa stellt ein mögliches Warnsymptom der Haut für lebensbedrohliche kardiovaskuläre Komplikationen wie Aortitis, Aortenaneurysma, Koronararterienstenose und Herzversagen dar [2, 4, 9, 10, 15, 18]. Während das Sweet-Syndrom des Erwachsenen narbenlos abheilt, findet sich bei einem Teil der Kinder eine residuale narbig-faltige Abheilung der Sweet-Läsionen [16]. Solch eine erworbene Cutis laxa ohne genetischen Hintergrund ist bislang bei 10 Kindern beschrieben worden, von denen auffälligerweise 6 Kinder Veränderungen an der Aorta (Aortitiden, Aneurysmen), den abgehenden großen Gefäßen und am Herzen aufwiesen [2, 4, 9, 10, 15, 18]. Wir berichten von einem Kind, bei dem das mit postinflammatorischer Elastolyse abheilende Sweet-Syndrom zur Diagnose einer Takayasu-Arteriitis führte.

## Anamnese und Befund

**Figure 1:**
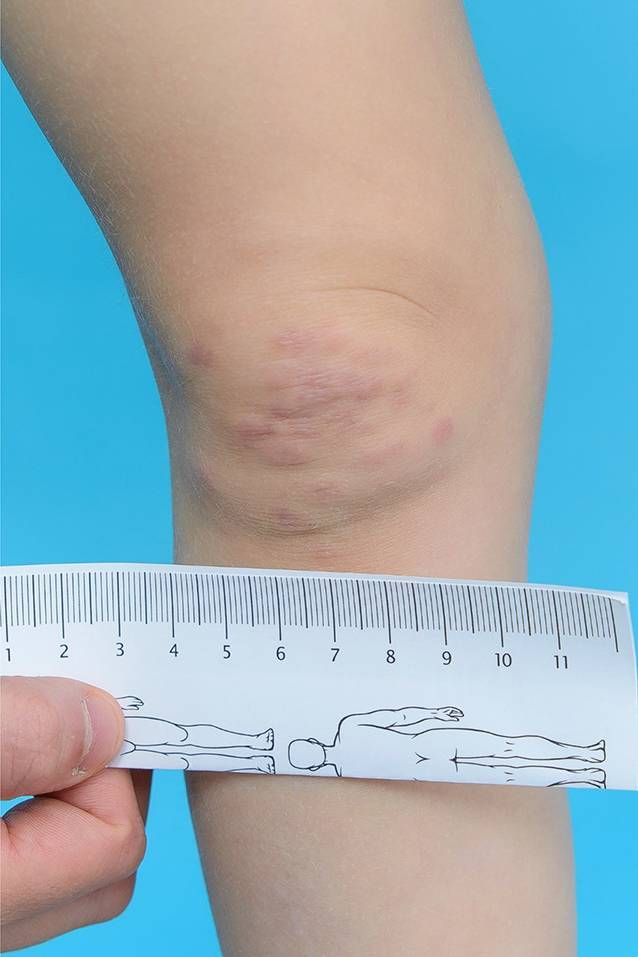

Eine 3‑jährige kaukasische, bisher gesunde Patientin wurde konsiliarisch von der Kinderklinik vorgestellt wegen seit 3 Monaten bestehender, asymmetrisch verteilter, druckschmerzhafter, erythematöser Papeln und Plaques im Gesicht und an den Gelenkstreckseiten der großen Extremitäten und Handrücken (Abb. 1). Im Verlauf entwickelten sich intermittierendes Fieber und Abgeschlagenheit, einhergehend mit dem Auftreten neuer Papeln.

## Histologie

**Figure 2:**
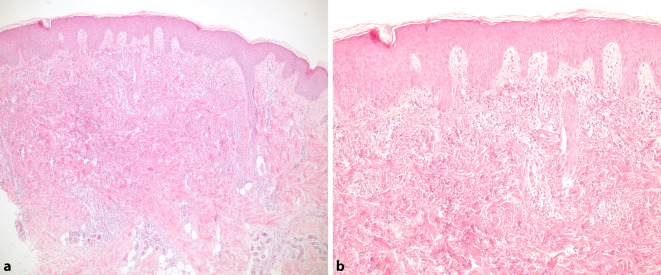

In der Stanzbiopsie vom Handrücken zeigten sich im Korium unter einem Ödem des Stratum papillare dichte diffuse Infiltrate aus vorwiegend neutrophilen Granulozyten durchmischt mit eosinophilen Granulozyten, Makrophagen und Lymphozyten (Abb. 2a, b).

## Labor

In den Laboruntersuchungen fielen erhöhte Entzündungsparameter mit einer Leukozytose von 12,5 G/l (Norm bis 12 G/l) und einer Neutrophilie (neutrophile Granulozyten absolut 8,04 G/l [Norm bis 7,28 G/l]), einer CRP-Erhöhung von 94,3 mg/l (Norm < 5 mg/l), einer Beschleunigung der BSG von 105/130 mm/h und eine Ferritinerhöhung auf 121,4 g/l (Norm bis 61 g/l) auf.

## Bildgebende Diagnostik

**Figure 3:**
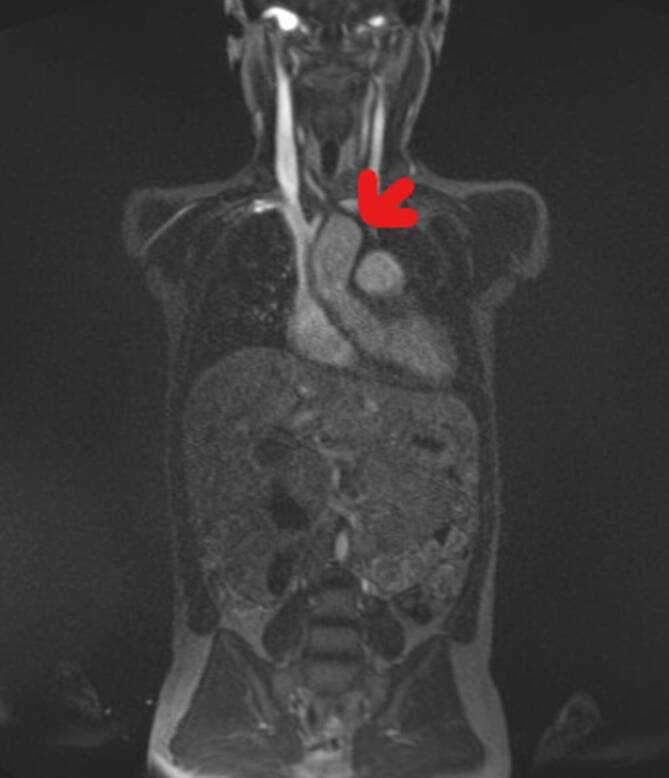

Die Sonographie des Mediastinums und das Angio-MRT, die wegen eines verbreiterten Herzschattens in der Thoraxröntgenaufnahme durchgeführt wurden, ergaben eine Aortitis mit deutlicher Verdickung und Kontrastmittel-vermittelter Signalverstärkung der Aortenwand, Ektasie der Aorta ascendens auf 22 mm (Norm < 10 mm) sowie Beteiligung der abgehenden supraaortalen Gefäße bis zur Karotisgabel, der Aorta pulmonalis und geringer Beteiligung der Viszeral- und Beckenarterien (Abb. 3).

## Diagnose

Sweet-Syndrom mit postinflammatorischer Elastolyse und erworbener Cutis laxa (Marshall-Syndrom) als Erstmanifestation einer Takayasu-Arteriitis im Kindesalter

## Therapie und Verlauf

**Figure 4:**
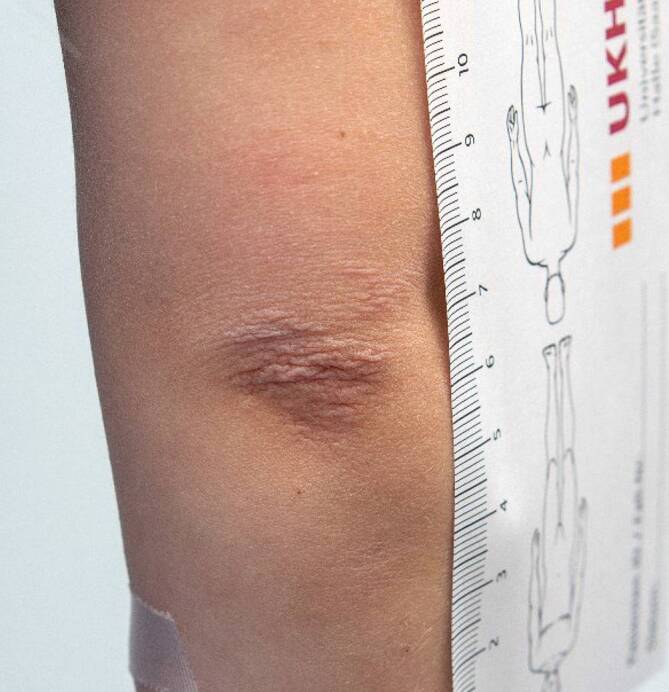

Neben der kardioprotektiven Medikation mit Acetylsalicylsäure (15 mg/Tag), Ramipril 2,5 mg und Propanolol 3‑mal 2,5 mg wurde eine immunsuppressive Therapie mit einem Methylprednisolon-Stoß über 3 Tage (30 mg/kgKG/Tag) eingeleitet, im Verlauf folgten tägliche Gaben von Prednisolon (1 mg/kgKG/Tag) in ausschleichender Dosierung, eine orale Therapie mit MTX 7,5 mg (Abbruch wegen Erbrechen) und eine Cyclophosphamid-Stoßtherapie (750 mg/m^2^) alle 3 Wochen für 3 Monate. Bei radiologischem Progress der Arteriitis der großen Arterien wurde die Therapie auf Adalimumab 20 mg subkutan umgestellt. Hierunter kam es zwar nach 3 Monaten zur Rückbildung der erythematösen Knoten unter Hinterlassung weicher Cutis-laxa-artiger Narben (Abb. 4), ein Rückgang der Arteriitis und Entzündungsparameter konnte allerdings erst unter der Therapie mit dem Interleukin-6-Antagonisten Tocilizumab mit 12 mg/kgKG (in Analogie zur Dosierung bei systemischer juveniler idiopathischer Arthritis) alle 2 Wochen i.v. erzielt werden.

## Diskussion und Review der Literatur

Das 1964 beschriebene Sweet-Syndrom, die akute neutrophile Dermatose, ist eine Erkrankung des Erwachsenenalters [22]. Nur 8 % aller Sweet-Syndrome treten im Kindesalter auf [19, 25]. Typischerweise finden sich wie bei unserer Patientin druckdolente Papeln und Plaques zusammen mit Fieber und weiteren Allgemeinsymptomen sowie laborchemische Neutrophilie. Während in der Erwachsenenpopulation vorwiegend Frauen mittleren Alters erkranken, besteht beim Sweet-Syndrom im Kindesalter keine Geschlechtsprädisposition bzw. leichte männliche Prädominanz mit einem Haupterkrankungsalter in den frühen Lebensjahren [11, 15]. Am häufigsten kommt im Kindesalter mit 42 % das mit transienten Atemwegs- und gastrointestinalen Infektionen einhergehende Sweet-Syndrom vor, gefolgt von sekundären parainflammatorischen, paraneoplastischen und medikamenteninduzierten Formen. Die häufigsten kindlichen assoziierten Neoplasmen sind akute myeloische Leukämie, Osteosarkome und myelodysplastische Syndrome [6, 25]. Schwangerschaftsassoziierte Formen sind im Kindesalter nicht relevant. Pathophysiologisch deutet u. a. die Erhöhung von Interleukin-1β auf eine Zuordnung des Sweet-Syndroms zu den autoinflammatorischen Erkrankungen hin [17].

Eine Besonderheit des Sweet-Syndroms des Kindesalters ist die fakultative Abheilung in Form einer postinflammatorischen Elastolyse mit erworbener Cutis laxa, die nach dem Erstbeschreiber als Marshall-Syndrom benannt ist [16]. Unter den 2 Typen der nichthereditären erworbenen Cutis laxa gehört sie zum Typ 2, d. h. der lokalisierten Cutis laxa. Der Typ 1 hingegen tritt schleichend generalisiert meist im Erwachsenenalter auf und kann mit systemischer Beteiligung (Emphyseme, gastrointestinale oder urogenitale Divertikel, Gefäßbeteiligung) einhergehen. Während der Typ 1 des Erwachsenenalters sekundär nach vorangegangenen entzündlichen (z. B. Urtikaria, Autoimmunerkrankungen, granulomatöse Erkrankungen, Amyloidose, Mastozytose), neoplastischen (lymphoproliferative Erkrankungen, Paraproteinämien), infektiösen Erkrankungen und medikamentenassoziiert (Penicillin, D‑Penicillamin, Isoniazid, SSRI) generalisiert auftritt [13, 24], manifestiert sich der Typ 2, das Marshall-Syndrom, häufiger bei Kindern lokalisiert und limitiert auf die präexistenten Effloreszenzen vorwiegend nach neutrophilenreicher Entzündung. Dem Verlust bzw. der Fragmentierung elastischer Fasern liegen hier wahrscheinlich exzessive Elastasefreisetzung aus neutrophilen Granulozyten und Makrophagen oder eine Dysfunktion von Elastaseinhibitoren zugrunde [5, 13, 17]. Dieses Phänomen ist besonders im Zusammenhang mit dem Sweet-Syndrom, dies auch im Rahmen einer Takayasu-Arteriitis, beschrieben.

## Takayasu-Arteriitis: dritthäufigste Vaskulitis des Kindesalters

Die Takayasu-Arteriitis (TA) ist eine seltene granulomatöse Entzündung der großen Gefäße, d. h. der Aorta und ihrer Hauptäste, unklarer Genese [21]. Die seltene TA im Kindesalter („childhood-onset Takayasu arteritis“ [c-TA]) ist nach der Purpura Schönlein-Henoch und dem Kawasaki-Syndrom die dritthäufigste Vaskulitis des Kindesalters. Die Diagnose der kindlichen TA beruht auf den EULAR/PRINTO/PRES-Klassifikationskriterien von 2006, die angiographische (konventionell/CT/MR) Gefäßwandveränderungen der Aorta oder ihrer Hauptäste als Majorkriterium mit mindestens einem Minorkriterium wie a) fehlender/reduzierter/ungleicher arterieller Puls oder Claudicatio, b) Diskrepanz des 4‑Extremitäten-Blutdrucks größer 10 mm Hg Differenz in einer Extremität, c) Gefäßgeräusche oder palpable Sensationen über den großen Gefäßen, d) systolischer/diastolischer RR > 95 % Perzentile, e) BSG > 20 mm Hg in der 1. Stunde oder erhöhtes CRP kombiniert (Tab. 1; [7]). Mit der charakteristischen Aortendilatation im Angio-MRT und der BSG- bzw. CRP-Erhöhung waren die Kriterien bei unserer Patientin erfüllt.

**Table 1:** 

Kategorie der Kriterien	Beschreibung
Obligates Majorkriterium	Angiographie (konventionell/CT/MRT) der Aorta oder ihrer Hauptäste und Pulmonalarterien: Aneurysma oder Dilatation, Stenosierung, Okklusion, verdickte Arterienwand (nicht durch fibromuskuläre Dysplasie o. Ä.)
Minorkriterien	Eines von 5 der folgenden:
A	Verlust/Abfall/Ungleichheit peripherer arterieller Pulse oder Claudicatio: fokaler Muskelschmerz durch physische Aktivität
B	Diskrepanz des 4‑Extremitäten-systolischen Blutdrucks > 10mm Hg Differenz in einer Extremität
C	Hörbare Geräusche oder palpatorische Sensation über großen Arterien
D	Systolischer RR/diastolischer RR > 95 % Körpergrößenperzentile
E	BSG > 20 mm in der 1. Stunde oder C‑reaktives Protein oberhalb der Norm

*RR* Blutdruck, *BSG* Blutsenkungsgeschwindigkeit

Pathophysiologisch führen transmurale granulomatöse Entzündungsinfiltrate zu Gefäßumbauprozessen mit Wandverdickung, Fibrose, Stenose und thrombotischer Okklusion sowie aneurysmatischer Dilatation [4]. Zusätzlich zur Gefäßentzündung kann die TA mit extravaskulären Erkrankungen einhergehen. Obwohl die TA die einzige systemische Vaskulitis ist, für die bislang keine kutane Vaskulitis oder Fortleitung in die Hautgefäße beschrieben wurde [20, 21], finden sich in 2,8–28 % assoziierte kutane Manifestationen wie Erythema-nodosum- und Erythema-induratum-ähnliche Läsionen [3]. Aus dem Spektrum der autoinflammatorischen neutrophilen Erkrankungen wurden Pyoderma-gangraenosum-artige Ulzerationen und Sweet-Syndrome beobachtet [3].

Der vorliegende Fall beschreibt ein Sweet-Syndrom des Kindesalters mit postinflammatorischer Elastolyse und erworbener Cutis laxa als Erstdiagnose einer kindlichen Takayasu-Arteriitis. In der PubMed-Literaturrecherche finden sich – unseren Fall eingeschlossen – 11 Kasuistiken von Kindern mit derartiger Hautmanifestation (kongenitale Elastolysen bei z. B. α_1_-Antitrypsin-Mangel wurden nicht inkludiert [8, 12]), von denen 7 Kinder kardiovaskuläre Komplikationen in Form von Aortitis bzw. Aortendilatation und Stenosen der Koronararterien zeigten, 3 davon nahmen einen tödlichen Verlauf (Tab. 2). Eine c‑TA wurde explizit bei 3 Kindern diagnostiziert [2, 15] und war bei 3 von 4 weiteren, z. T. vor der Chapel Hill Consesus Conference (CHCC) publizierten Fallberichten zumindest wahrscheinlich, da hier bei Vorliegen einer Aortitis bzw. Aortendilatation ([4, 9, 10, 18]; Tab. 2) 3 in der histopathologischen Untersuchung intramurale entzündliche Infiltrate mit neutrophilen Granulozyten aufwiesen und 2 davon auch Granulome [10, 15, 18]. Es ist also möglich, dass bei allen Kindern eine TA nach heutiger Definition (CHCC 2012) vorgelegen hat.

**Table 2:** 

Erstautor, Jahr	Alter/Geschlecht bei Sweet-Syndrom	Kardiovaskuläre Manifestation	Zeit nach Sweet-Diagnose	Explizite Diagnose einer TA	Histologie der Gefäße	Ausgang zur Zeit des Berichtes
Heyl, 1971 [10]	8 J, w	Aortitis, ventrikuläre Hypertrophie, Myokardinfarkt	Nicht bekannt	Nein, aber Kriterien wie bei TA	Intramurale lymphozytäre und neutrophile Infiltrate und Granulome	Tod durch Myokardinfarkt mit Herzversagen
Muster, 1983 [18]	16 Mo	Aortitis, Aortenaneurysma, Herzversagen durch Obliteration der Koronararterien	13 Mo	Nein, aber Aortitis, Fieber, BSG-Erhöhung	Infiltrat aus neutrophilen Granulozyten, Koronarstenose	Tod durch Herzversagen
Christensen, 1983 [4]	17 Mo, w	Aortitis, Aortenaneurysma, Linksherzhypertrophie	1 J	Nein, aber Aortitis	Leuko- und lymphozytäres Infiltrat	Tod durch Herzversagen
Guia, 1999 [9]	9 Mo, m	Aortenaneurysma, Aneurysmen und Stenosen großer Gefäße und Koronargefäße	7 J	Nein (Angiokardiographie, Herzkatheter)	Nicht berichtet	Lebend, rheologische und diuretische Therapie
Bi, 2008 [1]	3 J, m	Nein	–	–	–	–
Timmer-DE, 2009 [23]	8 Mo	Nein	–	–	–	–
Campos, 2005 [2]	10 Mo, w	Aortenaneurysma, Stenose abgehender Gefäße	7 Mo	Ja (MRT und EULAR/PRINTO/PRES-Kriterien)	Nicht berichtet	Lebend, immunsuppressive Therapie
Ma, 2012 [15]	22 Mo, m	Aortitis, Aortenaneurysma	8 J	Ja	Gemischtzellige Infiltrate, kleines Granulom	Lebend, operative und immunsuppressive Therapie
Loyal, 2018 [14]	15 Mo, m	Nein	–	–	–	–
Jagati, 2019 [13]	3 J, w	Nein	–	–	–	–
Eigener Fall, 2019	3 J, w	Aortitis, Aortenaneurysma	Zeitgleich	Ja (EULAR/PRINTO/PRES-Kriterien)	Nicht berichtet	Lebend, immunsuppressive Therapie

*w* weiblich, *m* männlich, *Mo* Monate, *J* Jahre, *TA* Takayasu Arteriitis, *BSG* Blutsenkungsgeschwindigkeit

Soweit Angaben existierten, gingen – anders als in unserem Fall – alle diese Sweet-Syndrome den kardiovaskulären Manifestationen über einen längeren Zeitraum von bis zu 8 Jahren voraus (Tab. 2). Welcher erworbene (auto)inflammatorische Prozess zu der parallelen Entzündung von Haut und Gefäßwänden mit Elastolyse führte – eine Systemmanifestation der neutrophilen autoinflammatorischen Erkrankung oder ein vaskulitisches Geschehen –, lässt sich aus diesen wenigen Fällen nicht ableiten.

Therapeutisch kommen bei der Takayasu-Arteriitis immunsuppressive und -modulatorischen Ansätze mit systemischen Glukokortikosteroiden, Immunsuppressiva (Azathioprin, Methotrexat, Mycophenolat-Mofetil, Cyclosporin oder Cyclophosphamid) und Drittlinienoptionen wie TNF-α-Antagonisten (Infliximab, Adalimumab, Etanercept) oder Interleukin-6-Rezeptor-Antagonisten (Tocilizumab) zum Einsatz [7]. Auch bei unserer Patientin konnte nach systemischer intravenöser und oraler Steroidtherapie sowie Cyclophosphamid-Stoßtherapie und Behandlung mit Adalimumab erst der Einsatz von Tocilizumab einen Rückgang der Entzündungsaktivität bewirken.

## Fazit

- Eine besondere Variante des Sweet-Syndroms des Kindesalters ist die Abheilung in Form einer postinflammatorischen Elastolyse mit Cutis laxa.
- Die Cutis laxa ist ein Warnzeichen für die lebensbedrohlichen kardialen Komplikationen wie Aortitiden (wie der Takayasu-Arteriitis), Aortenaneurysmen, Koronararterienstenosen und Herzversagen.
- Das Sweet-Syndrom kann der kardialen Manifestation vorausgehen, oder wie in unserem Fall eine Erstmanifestation darstellen.
- Betroffene Kinder mit Sweet-Syndrom und postinflammatorischer Elastolyse sollten zeitnah kardiologisch und rheumatologisch untersucht und werden, um bei vaskulärer Beteiligung einen komplikativen Verlauf durch frühe antiinflammatorische und immunmodulierende Systemtherapie zu verhindern.
